# Investigating Potential Dose–Response Relationships between Vitamin D Status and Cognitive Performance: A Cross-Sectional Analysis in Middle- to Older-Aged Adults in the Busselton Healthy Ageing Study

**DOI:** 10.3390/ijerph19010450

**Published:** 2021-12-31

**Authors:** Janis D. Harse, Kun Zhu, Romola S. Bucks, Michael Hunter, Ee Mun Lim, Brian R. Cooke, John P. Walsh, Kevin Murray

**Affiliations:** 1 School of Population and Global Health, University of Western Australia, Perth 6009, Australia; michael.hunter@uwa.edu.au (M.H.); kevin.murray@uwa.edu.au (K.M.); 2 Department of Endocrinology and Diabetes, Sir Charles Gairdner Hospital, Perth 6009, Australia; kun.zhu@uwa.edu.au (K.Z.); eemun.lim@health.wa.gov.au (E.M.L.); john.walsh@health.wa.gov.au (J.P.W.); 3 Discipline of Internal Medicine, Medical School, University of Western Australia, Perth 6009, Australia; 4 School of Psychological Science, University of Western Australia, Perth 6009, Australia; romola.bucks@uwa.edu.au; 5 Busselton Population and Medical Research Institute, Busselton 6280, Australia; 6 PathWest Laboratory Medicine, Department of Clinical Biochemistry, Queen Elizabeth II Medical Centre, Perth 6009, Australia; 7 PathWest Laboratory Medicine, Department of Clinical Biochemistry, Fiona Stanley Hospital, Perth 6150, Australia; Brian.Cooke@health.wa.gov.au

**Keywords:** vitamin D, 25-hydroxyvitamin D, cognitive performance, cognitive ageing, global cognition, domain-specific cognition

## Abstract

Low vitamin D status has been linked to adverse cognitive outcomes in older adults. However, relationships at higher levels remain uncertain. We aimed to clarify patterns of association between vitamin D status and cognitive performance, using flexible regression methods, in 4872 middle- to older-aged adults (2678 females) from the Busselton Healthy Ageing Study. Cross-sectional associations of serum levels of 25-hydroxyvitamin D (25OHD) and performance in cognitive domains were modelled using linear regression and restricted cubic splines, controlling for demographic, lifestyle, and health factors. Mean ± SD serum 25OHD levels were 78 ± 24 nM/L for women and 85 ± 25 nM/L for men. Increasing levels in women were associated with better global cognition (linear trend, *p* = 0.023) and attention accuracy (continuity of attention), with improvement in the latter plateauing around levels of 80 nM/L (nonlinear trend, *p* = 0.035). In men, increasing levels of serum 25OHD were associated with better attention accuracy (linear trend, *p* = 0.022), but poorer semantic verbal fluency (linear trend, *p* = 0.025) and global cognition (nonlinear trend, *p* = 0.015). We identified patterns of association between serum 25OHD levels and cognitive performance that may reflect early dose–response relationships, particularly in women. Longitudinal analyses extending through to older ages may help to clarify the nature, strength, and temporality of these relationships.

## 1. Introduction

Low levels of circulating 25-hydroxyvitamin D (25OHD), the standard measure of vitamin D, have been consistently associated with poorer health outcomes including cognitive decline in older adults [1]. The relationship with cognitive decline is most often reported in relation to global cognition [2,3,4,5] but is also noted for specific cognitive domains such as executive function [6,7,8,9] and attention and processing speed [3,4,9,10]. It is less evident for episodic and verbal memory [1,6,11]. The relationship remains unclear for higher circulating levels of 25OHD, with better performance [2,3,4,6,8,9,10,12], plateauing of performance [7,13,14,15], and poorer performance [16,17,18] all having been reported. The existence of nonlinear dose–response relationships may help explain mixed results from intervention studies, as no benefit could be expected from supplementing individuals with already adequate or high baseline levels.

Biological studies suggest that vitamin D, in its active hormonal form, has a neuroprotective role in the brain [19,20]. Receptors for the active, hormonal form of vitamin D, 1,25 dihydroxy-vitamin D (1,25(OH)_2_D), have been mapped in neuronal and glial cells [21]. Along with maintaining intraneuronal calcium levels, vitamin D regulates the production of neurotrophic factors required for nerve cell growth and the release of neurotransmitters such as acetylcholine, dopamine, and serotonin [19]. It is also known to modulate inflammatory, immune, and oxidative pathways [22], some of which are associated with neuronal ageing. In vitro, 1,25(OH)_2_D stimulates phagocytosis and clearance of the beta-amyloid material that is a hallmark of Alzheimer’s disease (AD) [23]. Nonlinear dose dependencies have also been demonstrated, with physiological levels conferring protection from excitotoxic insults that low and high levels do not [24,25].

There is medical agreement that 25OHD levels below 50 nM/L are inadequate for bone health [26]; in addition, vitamin D deficiency has been linked to numerous other adverse health outcomes including cardiovascular disease [27,28], cancers [29], stroke [27], and all-cause mortality [30,31]. Consensus is still lacking as to what constitutes optimal vitamin D status, reflecting the lack of understanding of the relationship across the full range of 25OHD levels. Traditional, analytic approaches in epidemiological research of categorising vitamin D or treating it as a simple, continuous variable are not well-suited to characterising patterns of association across the range. Contemporary regression methods, in which segments (splines) of the range are modelled separately, offer greater precision and flexibility [32]. To date, they have been applied in a limited way to vitamin D and cognition, mostly as an adjunct to traditional methods. In two European studies, the application of restricted cubic splines (RCS) identified nonlinear patterns in relation to global cognition [14,15] and attention and processing speed [15]. The relationship between low serum 25OHD levels and poorer performance was reinforced while, at higher levels, performance plateaued. However, both cohorts had relatively low serum 25OHD levels such that the capacity to explore the relationship at high levels may have been limited. Conversely, in two non-European studies where serum 25OHD levels were higher, power was likely compromised by small study sizes (*N* < 200); a positive association plateauing at around 120 nM/L was reported in relation to performance on an executive function task [8], while a negative association was reported for an episodic memory task, with the nonlinear model not improving upon the linear one [18].

Our study was based on a large community cohort of middle- to older-aged adults where serum 25OHD levels were known to be relatively high. We aimed to systematically investigate patterns of association between vitamin D status and performance across several cognitive domains, using linear regression and restricted cubic spline analysis. We hypothesised that patterns would be nonlinear, and their reflection of potential dose–response relationships could have important implications for health promotion policy and supplementation practice.

## 2. Materials and Methods

### 2.1. BHAS Study Cohort

The shire of Busselton, in the southwest region of Western Australia, has been the site of regular health surveys since 1966. The longitudinal Busselton Healthy Ageing study (BHAS) commenced in 2010 with the aim of characterising the physical and cognitive ageing of ‘baby-boomers’ within the community [33]. All residents of the City of Busselton’s Local Government Area (LGA) born between 1946 and 1964 were identified from the compulsory Western Australian electoral roll and invited to participate (*N* = 8223). A total of 82% were able to be contacted and confirmed as eligible (noninstitutionalised and still living in the region), and 76% of those contacted (*N* = 5107) agreed to take part in the baseline assessment. All participants completed a comprehensive health and risk factor questionnaire and attended the Health Survey Centre for physical and cognitive assessments between 2010 and 2015. Ethics approval was granted from the University of Western Australia Human Research Ethics Committee (Number RA/4/1/2203).

### 2.2. Serum 25-Hydroxyvitamin D Measurement

The vitamin D status of BHAS participants was determined from fasting blood samples collected at the time of survey centre visits. Serum 25OHD levels were assayed from these samples using the Abbott ARCHITECT platform (Abbott Laboratories, Abbott Park, IL, USA), a chemiluminescent method. The inter-assay coefficient of variation was 4.0% at 57.5 nmol/L and 2.6% at 178.3 nmol/L. A subsample (*n* = 117), randomly selected within three strata of 25OHD, were also assayed using the reference method of liquid chromatography–tandem mass spectrometry (LC–MS/MS), in accordance with published methods [34]. There was excellent agreement between the two methods (*r* = 0.94). Both assays are included in national external quality assurance programs.

### 2.3. Cognition

Cognitive function was assessed using the Cognitive Drug Research (CDR) computerised assessment system (Bracket Global, Reading, UK) [35]. This set of tasks assesses immediate and delayed word recall and recognition, simple and choice reaction time, digit vigilance, spatial and numeric working memory, and delayed picture recognition. Five summary scores were derived through a previously established factor analysis [36]. Accuracy components of the choice reaction time and digit vigilance tasks contribute to the continuity of attention factor, while the time taken to complete these and the simple reaction time task contribute to the power of attention factor. Likewise, quality of episodic memory, quality of working memory, and speed of memory factors were derived from accuracy and speed components of the memory, recall, and recognition tasks. Better performance was reflected by higher scores in all factors except power of attention and speed of memory, which are based on times taken to complete tasks; thus, better performance is reflected by lower scores.

In addition, pencil-and-paper tests assessed semantic and letter verbal fluency, covering domains of language and executive function, and the Mini-Mental State Examination (MMSE) [37] was used to assess global cognitive function. The National Adult Reading Test (NART-2) [38] was also administered to provide an estimate of IQ for use as a covariate in the analysis. NART assesses the ability to pronounce words that are spelt irregularly, which is an aspect of cognition that is usually well-preserved with age and injury-related changes to the brain.

### 2.4. Covariates

All covariates were selected based on their potential to confound the analysis. While most were identified from the literature, both employment status and hours spent sitting per day were included when preliminary analysis suggested they were associated with both serum 25OHD level and cognitive performance in our study cohort. Covariate information was derived from the BHAS questionnaire or from assessments undertaken at the survey centre.

Demographic factors were age, sex, estimated IQ, and current employment status. The latter was categorised as ‘retired’, ‘employed’, or ‘other’ (including being unemployed, being unable to work due to illness or disability, looking after the home and family, or doing voluntary work).

Lifestyle factors were body mass index (BMI), alcohol consumption, smoking status, physical activity, hours spent sitting per day, and the use of vitamin D supplements. Both BMI, calculated as weight (kilograms) divided by height squared (metres), and hours spent sitting per day were treated as continuous variables. Smoking status was categorised as ‘never smoked’, ‘ex-smoker’, ‘current smoker of fewer than 15 cigarettes per day’, or ‘current smoker of 15 or more cigarettes per day’. Alcohol consumption and physical activity were categorised after preliminary analysis suggested their relationships with the cognitive outcomes were not linear. Alcohol consumption was categorised as ‘nil’ or within quartiles of ‘0.1 to 2.5’, ‘2.6 to 8.5’, ‘8.6 to 17.9’, and ‘18+’ glasses per week. Physical activity was categorised as ‘low’, ‘medium’, or ‘high’ in accordance with scoring guidelines for the International Physical Activity Questionnaire (IPAQ) short form [39].

Other health factors and comorbidities included self-reported general health status (poor, fair, good, very good, or excellent), which was treated as a categorical variable. The presence or history of individual comorbidities including cardiovascular disease (CVD), hypertension, diabetes, depression, and anxiety was treated as a binary variable. Depression was indicated by a self-reported history of any doctor-diagnosed and treated episode of depression or a score above the standard cut-off in the nine-item Patient Health Questionnaire (PHQ-9) [40]. Anxiety was indicated from scores above the standard cut-off on either the Generalised Anxiety Disorder (GAD-7) assessment [41] or the Depression Anxiety Stress Score (DASS-21) [42].

### 2.5. Statistical Analysis

Data were excluded for four participants who self-reported diagnoses of vascular dementia or Alzheimer’s disease. Further exclusions were based on missing information for serum 25OHD level (*N* = 30), any cognitive test scores (*N* = 110), or any covariates (*N* = 92). Our final sample comprised 4872 participants, representing 95.4% of the baseline BHAS cohort. 

The vitamin D status of individual BHAS participants was measured from blood samples collected at different times of year, and circulating 25OHD levels are known to vary markedly with season. We used previously described methods to remove this seasonal effect [43,44], fitting a sinusoidal model to serum 25OHD levels with week of attendance as the predictor variable. The residual value in relation to each participant was added to the mean overall serum 25OHD level to obtain a predicted, de-seasonalised vitamin D status for each participant. 

To facilitate comparison across domains, we also standardised cognitive *z*-scores by subtracting the mean cohort scores from individual raw cognitive scores and dividing by the cohort standard deviation. IQ was estimated from the NART score, adjusting for age, sex, and years of education, in accordance with a method developed and validated in an Australian context [45]. All analyses were stratified by sex because the relationship between vitamin D and cognition differs between women and men [1].

Univariate analysis was initially performed for de-seasonalised 25OHD levels, cognitive outcomes, and all covariates, with means and standard deviations calculated for continuous variables, and frequencies calculated for categorical variables. The main analysis involved regression modelling to examine patterns of association between serum 25OHD levels and cognitive *z*-scores. Firstly, unadjusted, linear models were developed for each cognitive *z*-score in which serum 25OHD was treated as a simple, continuous term. We then developed four alternative, nonlinear, unadjusted models for each cognitive *z*-score, by applying restricted cubic spline (RCS) functions to serum 25OHD with three, four, five, or six knots located at recommended percentiles [32]. Akaike information criteria (AIC) were calculated with the lowest AIC identifying the most parsimonious, nonlinear model for each cognitive *z*-score (see Table S1). The likelihood ratio test was then applied to determine whether the selected RCS models improved upon the linear ones. Once the nonlinear or linear model was selected as the ‘best fit’ for each cognitive score, various levels of adjustment were applied such that four models were created for each cognitive *z*-score: 

Model 1: De-seasonalised 25OHD only;

Model 2: Model 1 plus age and estimated IQ;

Model 3: Model 2 plus BMI, alcohol consumption, smoking status, physical activity category, hours spent sitting per day, employment status, and the use of vitamin D supplements;

Model 4: Model 3 plus self-reported level of general health and presence or histories of diabetes, hypertension, CVD, anxiety, and depression.

We plotted the residuals for each model to check for violations of model assumptions. Least square mean cognitive *z*-scores with 95% confidence intervals were determined across the range of serum 25OHD from the fully adjusted, ‘best fit’ models. In graphical presentation, the *x*-axis was restricted to values of serum 25OHD between 30 nM/L and 150 nM/L, where 98% of values lay. To aid with interpretation, we also calculated and tabulated mean cognitive *z*-scores and 95% confidence intervals at mid-quartile levels of serum 25OHD for all models. 

Finally, a sensitivity analysis was performed, whereby participants reporting use of vitamin D supplements were excluded. In all analyses, *p*-values < 0.05 in two-tailed tests were considered statistically significant. SAS software, version 9.4 (SAS Institute Inc., Cary, NC, USA) was used for the preparation of data and to produce descriptive statistics. All regression models were developed, and graphical and tabular output was obtained in R version 4.0 (R Core Team, R Foundation for Statistical Computing, Vienna, Austria).

## 3. Results

### 3.1. Characteristics of the Study Cohort

The characteristics of women and men in our study cohort are presented in Table 1. Women made up 55% of the cohort, and mean age was similar for both sexes. Serum 25OHD levels were lower in women, and vitamin D deficiency was more prevalent, although more women reported the use of supplements. Levels of physical activity and alcohol consumption were both higher in men. A further breakdown of cohort characteristics by serum 25OHD quartiles is shown in Table S2.

### 3.2. Patterns of Association between Serum 25OHD Level and Cognitive Performance

**Figure 1:**
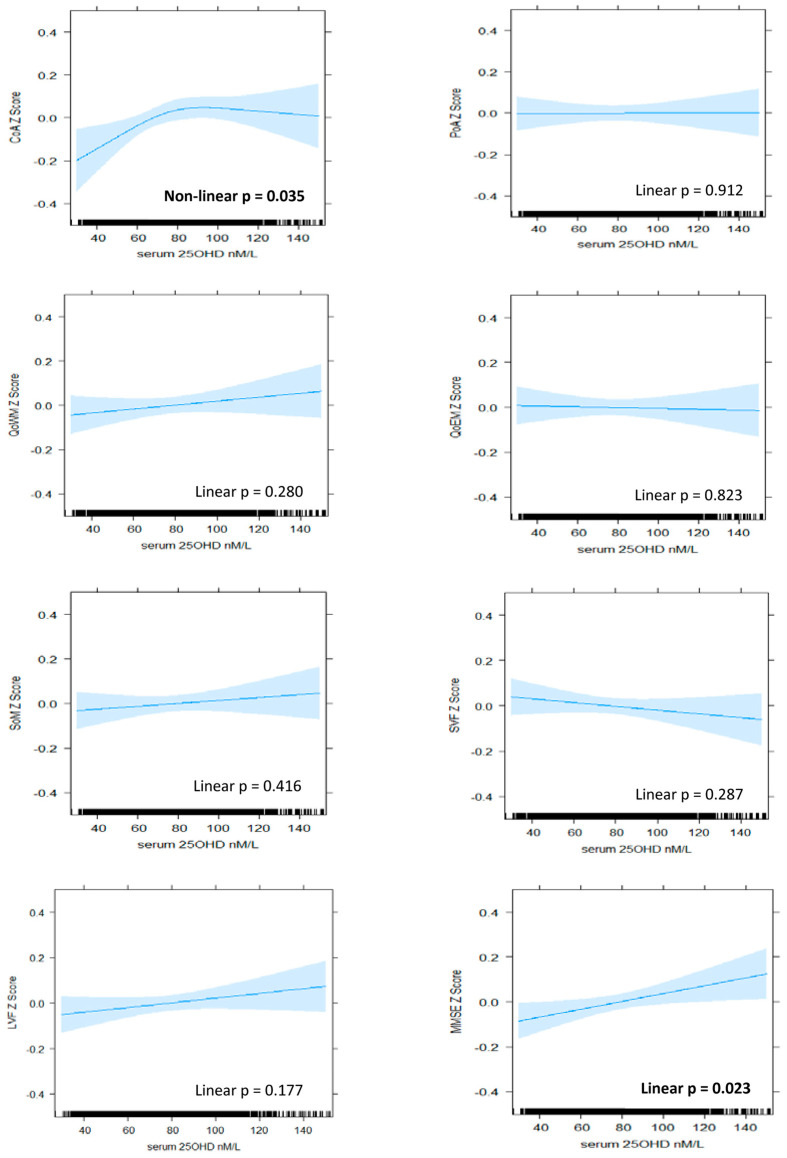
The association between serum 25OHD level (nM/L) and cognitive *z*-scores in BHAS women. Plots derived from ‘best fit’ of nonlinear (RCS) versus linear and fully adjusted models (Model 4). Model 4 includes de-seasonalised serum 25OHD level, age, estimated IQ, BMI, alcohol consumption, smoking status, physical activity, sitting hours per day, employment status, self-reported use of vitamin D supplements, self-reported health status, individual histories of hypertension, cardiovascular disease, diabetes, depression, and anxiety. CoA, continuity of attention; PoA, power of attention; QoWM, quality of working memory; QoEM, quality of episodic memory; SoM, speed of memory; SVF, semantic verbal fluency; LVF, letter verbal fluency; MMSE, Mini-Mental State Examination.

**Figure 2:**
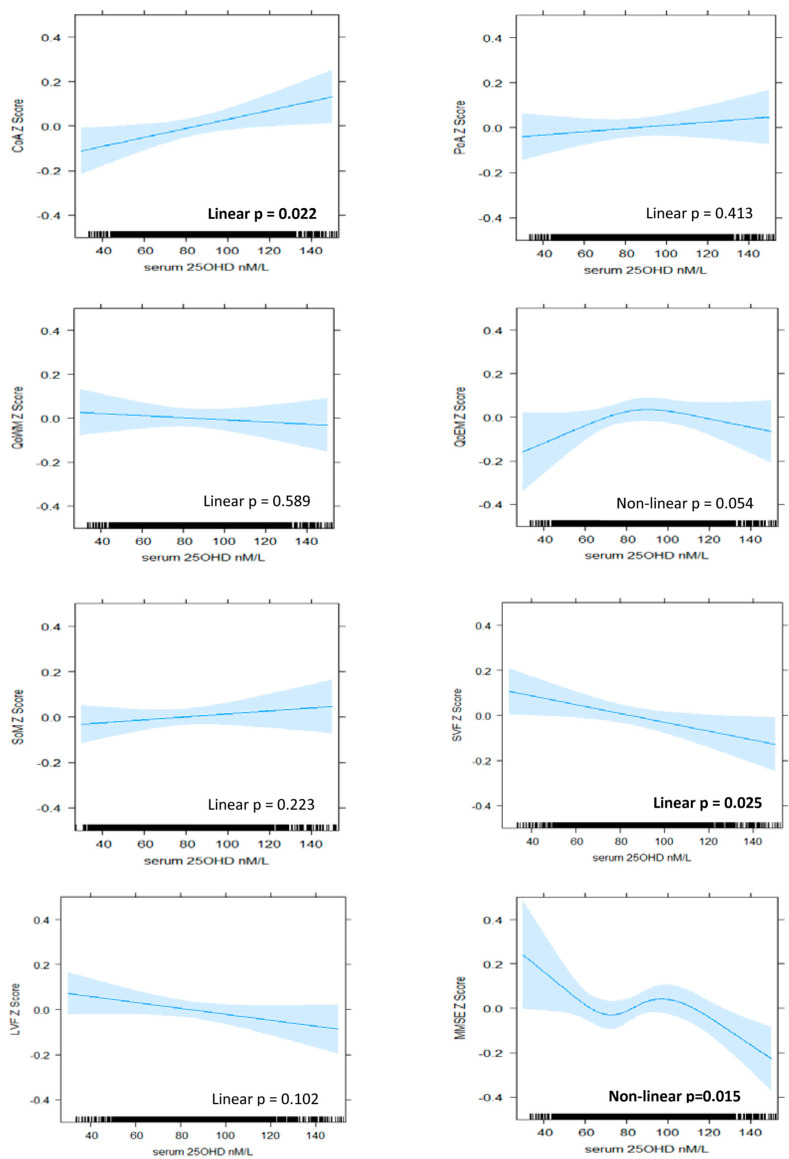
The association between serum 25OHD level (nM/L) and cognitive *z*-scores in BHAS men. Plots derived from ‘best fit’ of nonlinear (RCS) versus linear and fully adjusted models (Model 4). Model 4 includes de-seasonalised serum 25OHD level, age, estimated IQ, BMI, alcohol consumption, smoking status, physical activity, sitting hours per day, employment status, self-reported use of vitamin D supplements, self-reported health status, individual histories of hypertension, cardiovascular disease, diabetes, depression, and anxiety. CoA, continuity of attention; PoA, power of attention; QoWM, quality of working memory; QoEM, quality of episodic memory; SoM, speed of memory; SVF, semantic verbal fluency; LVF, letter verbal fluency; MMSE, Mini-Mental State Examination.

Results of the regression modelling are presented in Figure 1 and Figure 2, as well as Table 2 and Table 3. The plotting of residuals did not suggest any major violations of model assumptions. Nonlinear and linear patterns of association were identified between serum 25OHD level and cognitive performance in several, but not all, domains. Patterns varied by domain and between BHAS women and men. 

#### 3.2.1. Attention

A positive and nonlinear pattern of association was identified between serum 25OHD level and continuity (accuracy) of attention in women, which persisted with adjustment for all covariates (model 4, nonlinear *p* = 0.035). Improvements in performance were associated with increasing serum 25OHD up to levels of approximately 80 nM/L, above which the relationship plateaued (Figure 1). In men, a linear pattern was identified (model 4, linear *p* = 0.022), suggesting that improvements in accuracy of attention were associated with increasing serum 25OHD levels throughout the range (Figure 2). From the lowest to highest serum 25OHD quartiles, mean continuity of attention *z*-scores increased by approximately 0.13 SD in women and 0.09 in men (Table 2 and Table 3). This equated to less than one point in the raw continuity of attention factor scores for both sexes. No significant associations were observed between serum 25OHD levels and the power (speed) of attention, in either sex.

#### 3.2.2. Memory 

A positive and nonlinear pattern was identified between serum 25OHD level and quality of episodic memory in men in the unadjusted model (nonlinear *p* = 0.032). However, this was no longer statistically significant after adjustment for all covariates (model 4, nonlinear *p* = 0.054).

For both sexes, linear patterns were observed between serum 25OHD level and speed of memory retrieval in unadjusted analyses (women, linear *p* = 0.032; men, linear *p* = 0.010), but these were also no longer significant after adjustment for age and estimated IQ (women, linear *p* = 0.150; men, linear *p* = 0.080). No association was identified between serum 25OHD level and quality of episodic memory in women or quality of working memory in either sex.

#### 3.2.3. Verbal Fluency

A negative, linear relationship was identified between serum 25OHD level and semantic fluency in men (model 4, linear *p* = 0.025), suggesting that poorer performance was associated with increasing levels of serum 25OHD (Figure 2). From the lowest to highest quartiles of serum 25OHD, mean semantic fluency decreased by 0.10 SD (Table 3), equating to less than one word. No significant patterns of association were identified for semantic fluency in women nor for letter fluency in either sex.

#### 3.2.4. Global Cognition 

A linear pattern of positive association was identified between serum 25OHD levels and MMSE score in women (model 4, linear *p* = 0.023). An improvement in mean MMSE *z*-score of 0.08 SD was associated with moving from the lowest to highest quartile (Table 3), which equated to less than 1 point on the raw MMSE score scale. In men, a nonlinear and overall negative pattern was identified (model 4, nonlinear *p* = 0.015). Better performance was associated with low levels of serum 25OHD, and poorer performance was associated with high levels, although, at mid-range levels (from 70 nM/L to 100 nM/L), the pattern reversed such that increasing serum 25OHD level was associated with improving performance (Figure 2).

### 3.3. Sensitivity Analysis

The removal of those reporting the use of vitamin D supplements from the analyses mildly enhanced the association between serum 25OHD level and continuity (accuracy) of attention in women (model 4, nonlinear *p* = 0.019) and both semantic fluency (model 4, linear *p* = 0.009) and quality of episodic memory in men (model 4, nonlinear *p* = 0.031). Conversely, associations with MMSE score were attenuated, more so in women (model 4, linear *p* = 0.092) than in men (model 4, nonlinear *p* = 0.016), (see Supplementary Tables S3 and S4).

## 4. Discussion

Our investigation of the relationship between vitamin D and cognitive performance in middle- to older-aged adults highlighted several nonlinear and linear patterns of association, although effect sizes were small. Associations were more positive in women, compared with men. This is consistent with the literature which suggests that the relationship between vitamin D and cognitive performance is stronger for women [1]. There are physiological interactions between the effects of vitamin D and oestrogen [22], such that the postmenopausal depletion of oestrogen may render women more vulnerable to the effects of vitamin D deficiency [46].

Accuracy of attention was the only domain that was positively associated with serum 25OHD levels in both sexes. Lower levels, below approximately 80 nM/L in women and 90 nM/L in men, were associated with poorer accuracy of attention. Patterns diverged at higher levels, with a plateauing effect observed for women and continued improvement observed for men. In the literature, a relationship between low vitamin D status and poorer performance in older adults has been reported with respect to attention and other specific domains, although thresholds have varied [1]. Likewise, a divergence of patterns at higher levels is common in the literature, although not necessarily based on sex. When the same continuity of attention factor was examined in a cohort of older adults (85 years plus), moderate levels of serum 25OHD were associated with better performance than lower levels, but not higher levels [13]. A plateauing pattern was also modelled for performance in a coding task that assessed attention and processing speed [15]. In contrast, mean performance was reported to improve throughout the range of serum 25OHD levels for both the digit symbol substitution test [3,10] and the digit span task [6], two other measures of attention and processing speed that also tap working memory.

We observed a significant association between vitamin D and global cognition (measured with the MMSE) for both sexes, but the patterns were very different. The positive and linear pattern identified for vitamin D status and global cognition in women has many precedents in the literature. Improved performance throughout the range was reported in mixed-sex studies using the MMSE [6,47], as well as other measures of global cognition such as the abbreviated mental test [5] and composite scores [3,4,48]. While nonlinear patterns have also been previously reported in relation to global cognition [14,15], they do not resemble the complex pattern identified in our study for men. This pattern is difficult to interpret and lacks biological plausibility as an overall dose–response pattern. While the positive association through mid-range levels of serum 25OHD and the association between higher levels and poorer performance are consistent with some previous findings [16,17], the association between lower vitamin D levels and better performance runs contrary to most of the literature. Nevertheless, there is some consistency with the negative association we also observed for semantic fluency in men. Furthermore, a negative and linear pattern of association was reported between serum 25OHD level and verbal learning and memory in another Western Australian study [18]. This latter cohort was slightly older (mean 66 years), and 25OHD levels were higher (mean = 85 nM/L) than in our cohort. A form of reverse causation was posited, whereby late middle-aged and healthy individuals were likely to be engaged in predominantly indoors, white collar work and have high cognitive function but low vitamin D status [18]. Three-quarters of the men in our cohort were still employed, and, while we adjusted for employment status and related factors such as sitting hours per day and estimated IQ, we were unable to adjust for occupation type. In addition, the variability in the lower part of the serum 25OHD range, where data are scarce, suggests further caution in the interpretation of this finding.

Our study had several strengths including its size and the use of RCS to systematically investigate the patterns of association between vitamin D status and cognitive performance across the range of serum 25OHD levels. Relatively few significant associations were identified, and effect sizes were small, suggesting the relationship between vitamin D and cognitive performance may not be strong. However, this may reflect both the relatively young age and high vitamin D status of our study cohort. The strongest associations have been previously reported where adults are over 65 years of age [1,49,50]. Similarly, findings are stronger in studies where vitamin D deficiency is more prevalent [6,51]. While middle age may be a critical time for maintaining adequate vitamin D status to protect the nervous system, the effects of depletion may not manifest until it becomes chronic in later life and there is vulnerability to other insults [52].

The pattern identified for accuracy of attention in women has particular biological plausibility with respect to the neuroprotective role suggested for vitamin D. The distinct decline in performance associated with lower serum 25OHD levels may represent an early, subclinical sign of vitamin D-related cognitive impairment in these middle- to older-aged women. In view of the normally long, preclinical phase associated with cognitive decline, identification of this marker may provide important opportunities for early intervention. Unlike many other potential risk factors, vitamin D deficiency is easily addressed through taking supplements.

The relatively high vitamin D status of our cohort facilitated an investigation of the relationship at higher levels. Yet, as with previous studies, our findings were mixed. In women, levels of at least 70–80 nM/L were associated with optimal cognitive performance, and higher levels were not associated with poorer performance in any domain. Our findings were less clear for men, with increasing vitamin D status associated with both positive and negative outcomes across different cognitive domains. Therefore, we cannot make any inference regarding vitamin D status and the cognitive health of middle- to older-aged men. While other factors may be at play, the negative relationships also suggest some caution with respect to vitamin D supplementation of men at this age.

Due to the cross-sectional nature of this study, we cannot be sure that the identified patterns of association reflect true dose–response relationships. While we excluded known cases of dementia, reverse causation could be responsible for the positive relationships observed between vitamin D and cognition. Older adults with poorer cognition may get less sunlight and have a poorer diet in comparison to those with better cognition. Additionally, while we adjusted for numerous factors, some residual confounding also appears likely. We did not adjust for occupation type and were also unable to distinguish between occupational and recreational physical activity, although the latter has been more positively associated with cognitive performance in older adults [53]. Some potential confounders such as smoking status and levels of alcohol consumption may have been underestimated as they relied on self-report.

While large randomised controlled trials may be required to address causation, they will need to be of long duration and should address the potential for nonlinear relationships. Further longitudinal studies should assess serum 25OHD levels and cognitive performance at multiple timepoints from middle through to older age to better understand the temporality of the relationship. The BHAS is a longitudinal study that should provide such opportunity in the future.

## 5. Conclusions

Our study identified positive patterns of associations between vitamin D status and attention accuracy in middle- to older-aged women and men, as well as global cognition in women. While causation was not established, these patterns do have some plausibility as causal, dose–response relationships. In particular, the sharp decline in attention accuracy associated with below average vitamin D status in women appears consistent with the neuroprotective role proposed in biological studies. While no inference can be made with respect to an optimal vitamin D status in middle- to older-aged men, a level of at least 70 to 80 nM/L was associated with better cognitive health in women at this age.

## Figures and Tables

**Table 1:** Main characteristics and raw cognitive scores for the study cohort.

	Women (*N* = 2678)	Men (*N* = 2194)
Age (years)	57.9 ± 5.7	58.1 ± 5.9
De-seasonalised serum 25OHD (nM/L)	78.3 ± 24.3	84.9 ± 24.6
Vitamin D deficient (<50 nM/L), *n* (%)	255 (9.5)	83 (3.8)
Estimated IQ	102.6 ± 9.6	102.1 ± 10.9
Body mass index (kg/m^2^)	27.9 ± 5.5	28.5 ± 4.1
Smoking status, *n* (%)		
Never	1350 (50.4)	931 (42.4)
Ex	1094 (40.8)	1014 (46.2)
Current <15 cigarettes per day	121 (4.5)	101 (4.6)
Current ≥15 cigarettes per day	113 (4.2)	148 (6.8)
Alcohol consumption (glasses per week), *n* (%)		
Nil	256 (9.6)	133 (6.1)
0 to 2.5	843 (31.5)	286 (13.0)
2.6 to 8.5	715 (26.7)	386 (17.6)
8.6 to 17.9	597 (22.3)	509 (23.2)
18+	267 (10.0)	880 (40.1)
Physical activity category (MET minutes/week), *n* (%)		
0–599	633 (23.6)	330 (15.0)
600–2999	1084 (40.5)	657 (30.0)
3000+	960 (35.9)	1207 (55.0)
Sitting hours per day	4.3 ± 2.5	4.7 ± 2.7
Employment status, *n* (%)		
Employed	1567 (58.5)	1603 (73.1)
Retired	680 (25.4)	427 (19.5)
Other	431 (16.1)	164 (7.5)
Use of vitamin D supplements, *n* (%)	435 (16.2)	135 (6.2)
Self-reported health status, *n* (%)		
Poor/fair	218 (8.1)	210 (9.6)
Good	986 (36.8)	907 (41.3)
Very good/excellent	1474 (55.1)	1077 (49.1)
Medical history, *n* (%)		
Depression	596 (22.3)	335 (15.4)
Anxiety	158 (5.9)	72 (3.3)
CVD	98 (3.7)	178 (8.1)
Diabetes	161 (6.0)	155 (7.1)
Hypertension	1023 (38.2)	988 (45.0)
Education (level completed), *n* (%)		
Primary or less	30 (1.1)	27 (1.2)
Secondary, including TAFE college	2109 (78.8)	1747 (79.6)
Tertiary	539 (20.1)	420 (19.1)
Raw cognitive scores		
Continuity of attention factor	90.6 ± 4.2	90.4 ± 3.8
Power of attention factor	1244.1 ± 138.4	1235.2 ± 139.4
Quality of working memory factor	187.7 ± 16.5	187.8 ± 15.2
Quality of episodic memory factor	190.1 ± 46.2	172.6 ± 44.7
Speed of memory factor	4319.6 ± 859.1	4359.9 ± 885.3
Semantic verbal fluency	19.0 ± 4.8	18.1 ± 4.6
Letter verbal fluency	40.1 ± 11.1	35.6 ± 10.8
MMSE	28.6 ± 1.6	28.4 ± 1.6

Values shown are means ± SD unless otherwise indicated; percentages (%) may not always add up to 100 due to rounding. TAFE, Technical and Further Education; MET, metabolic equivalent (a measure of physical activity related to metabolic rate); CVD, cardiovascular disease, MMSE, Mini-Mental State Examination.

**Table 2:** Cognitive *z*-scores (least square means and 95% confidence limits) at mid-quartile levels of serum 25OHD in BHAS women derived from ‘best fit’ models.

Cognitive Scores *	Model ^	Quartile 1 (53.0 nM/L)	Quartile 2 (68.6 nM/L)	Quartile 3 (82.9 nM/L)	Quartile 4 (104.2 nM/L)	*p*-Values #		Best Fit (Non Linear vs. Linear)
						Overall	Nonlinear	
Continuity of attention factor	1	−0.08 (−0.15, −0.02) ^a^	0.00 (−0.04, 0.05) ^b^	0.05 (−0.01, 0.10) ^c^	0.05 (0.00, 0.11) ^b,c^	**0.006**	**0.028**	Nonlinear 3 knots
	2	−0.10 (−0.16, −0.04) ^a^	0.00 (−0.04, 0.04) ^b^	0.05 (0.00, 0.10) ^c^	0.06 (0.01, 0.12) ^c^	**<0.001**	**0.009**	
	3	−0.08 (−0.15, −0.02) ^a^	0.00 (−0.04, 0.04) ^b^	0.05 (0.00, 0.09) ^c^	0.06 (0.00, 0.11) ^b,c^	**0.010**	**0.018**	
	4	−0.08 (−0.14, −0.01) ^a^	0.00 (−0.04, 0.04) ^b^	0.04 (−0.01, 0.09) ^c^	0.05 (0.00, 0.11) ^b,c^	**0.021**	**0.035**	
Power of attention factor	1	−0.01 (−0.07, 0.04)	−0.01 (−0.05, 0.04)	0.00 (−0.04, 0.04)	0.01 (−0.04, 0.07)	0.497	0.366	Linear
	2	0.00 (−0.05, 0.05)	0.00 (−0.04, 0.04)	0.00 (−0.04, 0.04)	0.00 (−0.05, 0.05)	0.970	0.182	
	3	0.00 (−0.05, 0.06)	0.00 (−0.04, 0.04)	0.00 (−0.08, 0.02)	0.00 (−0.06, 0.05)	0.842	0.101	
	4	0.00 (−0.05, 0.06)	0.00 (−0.04, 0.04)	0.00 (−0.04, 0.04)	0.00 (−0.06, 0.05)	0.912	0.128	
Quality of working memory factor	1	−0.03 (−0.08, 0.03)	−0.01 (−0.05, 0.03)	0.00 (−0.03, 0.04)	0.03 (−0.03, 0.08)	0.176	0.131	Linear
	2	−0.03 (−0.09, 0.02)	−0.01 (−0.05, 0.03)	0.01 (−0.03, 0.04)	0.03 (−0.02, 0.09)	0.085	0.078	
	3	−0.02 (−0.08, 0.03)	−0.01 (−0.05, 0.03)	0.00 (−0.03, 0.04)	0.02 (−0.03, 0.08)	0.262	0.096	
	4	−0.02 (−0.08, 0.03)	−0.01 (−0.05, 0.03)	0.00 (−0.03, 0.04)	0.02 (−0.03, 0.08)	0.280	0.121	
Quality of episodic memory factor	1	0.02 (−0.03, 0.08)	0.01 (−0.03, 0.05)	−0.01 (−0.04, 0.03)	−0.03 (−0.08, 0.03)	0.214	0.995	Linear
	2	0.01 (−0.04, 0.06)	0.00 (−0.04, 0.04)	0.00 (−0.04, 0.03)	−0.01 (−0.06, 0.04)	0.085	0.642	
	3	0.00 (−0.05, 0.06)	0.00 (−0.04, 0.04)	0.00 (−0.04, 0.04	0.00 (−0.06, 0.05)	0.883	0.447	
	4	0.00 (−0.05, 0.06)	0.00 (−0.04, 0.04)	0.00 (−0.04, 0.04)	−0.01(−0.06, 0.05)	0.823	0.440	
Speed of memory factor	1	−0.04 (−0.10, 0.01) ^a^	−0.02 (−0.06, 0.02) ^b^	0.01 (−0.03, 0.05) ^c^	0.04 (−0.01, 0.10) ^d^	**0.032**	0.736	Linear
	2	−0.03 (−0.08, 0.02)	−0.01 (−0.05, 0.03)	0.01 (−0.03, 0.04)	0.03 (−0.03, 0.08)	0.150	0.894	
	3	−0.01 (−0.07, 0.04)	−0.01 (−0.04, 0.03)	0.00 (−0.03, 0.04)	0.01 (−0.04, 0.07)	0.469	0.510	
	4	−0.02 (−0.07, 0.04)	−0.01 (−0.05, 0.03)	0.00 (−0.03, 0.04)	0.02 (−0.04, 0.07)	0.416	0.540	
		
Semantic verbal fluency	1	0.03 (−0.02, 0.09)	0.01 (−0.03, 0.05)	−0.01 (−0.05, 0.03)	−0.04 (−0.09, 0.02)	0.085	0.536	Linear
	2	0.02 (−0.03, 0.07)	0.01 (−0.03, 0.04)	0.00 (−0.04, 0.03)	−0.02 (−0.07, 0.03)	0.304	0.237	
	3	0.02 (−0.03, 0.07)	0.01 (−0.03, 0.05)	0.00 (−0.04, 0.03)	−0.02 (−0.08, 0.03)	0.283	0.183	
	4	0.02 (−0.03, 0.07)	0.01 (−0.03, 0.05)	0.00 (−0.04, 0.03)	−0.02 (−0.08, 0.03)	0.287	0.269	
Letter verbal fluency	1	−0.01 (−0.07, 0.04)	0.00 (−0.05, 0.04)	0.00 (−0.04, 0.04)	0.01 (−0.04, 0.07)	0.577	0.075	Linear
	2	−0.02 (−0.07, 0.03)	−0.01 (−0.05, 0.03)	0.00 (−0.03, 0.04)	0.02 (−0.03, 0.07)	0.216	0.071	
	3	−0.03 (−0.08, 0.03)	−0.01 (−0.05, 0.03)	0.00 (−0.03, 0.04)	0.02 (−0.03, 0.08)	0.200	0.062	
	4	−0.03 (−0.08, 0.02)	−0.01 (−0.05, 0.03)	0.00 (−0.03, 0.04)	0.03 (−0.03, 0.08)	0.177	0.058	
Mini-Mental State Examination	1	−0.03 (−0.09, 0.02)	−0.01 (−0.05, 0.03)	0.01 (−0.03, 0.04)	0.03 (−0.02, 0.09)	0.105	0.536	Linear
	2	−0.05 (−0.09, 0.00) ^a^	−0.02 (−0.05, 0.02) ^a^	0.01 (−0.03, 0.04) ^a^	0.05 (−0.01, 0.10) ^b^	**0.015**	0.787	
	3	−0.05 (−0.10. 0.00) ^a^	−0.02 (−0.06, 0.02) ^a^	0.01 (−0.03, 0.04) ^a^	0.05 (−0.00, 0.10) ^b^	**0.016**	0.991	
	4	−0.04 (−0.10, 0.01) ^a^	−0.02 (−0.05, 0.02) ^a^	0.01 (−0.03, 0.04) ^a^	0.04 (−0.01, 0.10) ^b^	**0.023**	0.814	

* Higher cognitive scores indicate better performance except in the case of power of attention and speed of memory, where higher scores indicate slower speed and worse performance. ^ Model 1: De-seasonalised serum 25OHD only; Model 2: Model 1 plus age and estimated IQ; Model 3: Model 2 plus BMI, alcohol consumption, smoking status, physical activity (low, medium, high), sitting hours per day, employment status (employed, retired or other), and use of vitamin D supplements; Model 4: Model 3 plus self-reported health status and history (yes vs. no) of hypertension, cardiovascular disease, diabetes, depression, and anxiety. # *p*-Value overall: overall *p*-value for serum 25OHD where nonlinear model was selected, or the *p*-value for the linear term where linear model was selected; *p*-value nonlinear: *p*-value from likelihood ratio test of whether nonlinear model improves on the simple, linear model. *p*-Values ≤ 0.05 are highlighted in bold. ^a–d^ In rows with superscripts (a, b, c, d), mean values without a common letter indicate that means differ, *p* < 0.05.

**Table 3:** Cognitive *z*-scores (least square means and 95% confidence limits) at mid-quartile levels of serum 25OHD in BHAS men derived from ‘best fit’ models.

Cognitive Scores *	Model ^	Quartile 1 (59.8 nM/L)	Quartile 2 (75.4 nM/L)	Quartile 3 (89.2 nM/L)	Quartile 4 (110.9 nM/L)	*p*-Values #		Best Fit (Non Linear vs. Linear)
						Overall	Nonlinear	
Continuity of attention factor	1	−0.04 (−0.10, 0.02)	−0.02 (−0.06, 0.03)	0.01 (−0.04, 0.05)	0.04 (−0.02, 0.10)	0.067	0.811	Linear
	2	−0.06 (−0.12, 0.00) ^a^	−0.02 (−0.07, 0.02) ^b^	0.01 (−0.03, 0.05) ^c^	0.06 (0.00, 0.12) ^d^	**0.007**	0.671	
	3	−0.05 (−0.11, 0.01) ^a^	−0.02 (−0.06, 0.02) ^b^	0.00 (−0.03, 0.05) ^c^	0.05 (−0.01, 0.11) ^d^	**0.021**	0.591	
	4	−0.04 (−0.11, 0.03) ^a^	−0.02 (−0.07, 0.02) ^b^	0.00 (−0.05, 0.05) ^c^	0.05 (−0.01, 0.11) ^d^	**0.022**	0.592	
Power of attention factor	1	−0.03 (−0.09, 0.03)	−0.01 (−0.06, 0.03)	0.00 (−0.04, 0.05)	0.03 (−0.03, 0.09)	0.195	0.455	Linear
	2	−0.01 (−0.07, 0.05)	0.00 (−0.05, 0.04)	0.00 (−0.04, 0.04)	0.01 (−0.05, 0.07)	0.656	0.366	
	3	−0.01 (−0.07, 0.04)	−0.01 (−0.05, 0.04)	0.00 (−0.04, 0.04)	0.02 (−0.05, 0.08)	0.510	0.306	
	4	−0.02 (−0.08, 0.04)	−0.01 (−0.05, 0.04)	0.00 (−0.04, 0.04)	0.02 (−0.04, 0.08)	0.413	0.274	
Quality of working memory factor	1	0.02 (−0.04, 0.08)	0.01 (−0.04, 0.05)	0.00 (−0.05, 0.04)	−0.02 (−0.08, 0.04)	0.401	0.518	Linear
	2	0.00 (−0.05, 0.06)	0.00 (−0.04, 0.05)	0.00 (−0.04, 0.04)	0.00 (−0.06, 0.06)	0.831	0.495	
	3	0.01 (−0.05, 0.07)	0.01 (−0.04, 0.05)	0.00 (−0.04, 0.04)	−0.01 (−0.07, 0.05)	0.574	0.379	
	4	0.01 (−0.05, 0.07)	0.00 (−0.04, 0.05)	0.00 (−0.04, 0.04)	−0.01 (−0.07, 0.05)	0.589	0.392	
Quality of episodic memory factor	1	−0.03 (−0.09, 0.04)	0.03 (−0.02, 0.07)	0.04 (−0.02, 0.09)	−0.01 (−0.07, 0.05)	0.084	**0.032**	Nonlinear 3 knots
	2	−0.04 (−0.11, 0.02) ^a^	0.02 (−0.03, 0.06) ^b^	0.04 (−0.02, 0.09) ^a,b^	0.01 (−0.04, 0.07) ^a,b^	0.108	**0.040**	
	3	−0.04 (−0.11, 0.02)	0.01 (−0.03, 0.06)	0.04 (−0.02, 0.09)	0.01 (−0.05, 0.07)	0.136	**0.050**	
	4	−0.04 (−0.10, 0.03)	0.02 (−0.03, 0.06)	0.04 (−0.02, 0.09)	0.01 (−0.05, 0.07)	0.151	0.054	
Speed of memory factor	1	−0.06 (−0.11, 0.00) ^a^	−0.02 (−0.07, 0.02) ^b^	0.01 (−0.03, 0.05) ^c^	0.06 (0.00, 0.12) ^d^	**0.010**	0.269	Linear
	2	−0.04 (−0.09, 0.02)	−0.01 (−0.06, 0.03)	0.01 (−0.03, 0.05)	0.04 (−0.02, 0.10)	0.080	0.202	
	3	−0.02 (−0.08, 0.03)	−0.01 (−0.05, 0.03)	0.01 (−0.04, 0.05)	0.03 (−0.03, 0.09)	0.249	0.200	
	4	−0.03 (−0.09, 0.03)	−0.01 (−0.05, 0.03)	0.01 (−0.04, 0.05)	0.03 (−0.03, 0.09)	0.223	0.196	
Semantic verbal fluency	1	0.06 (0.00, 0.12) ^a^	0.02 (−0.02, 0.07) ^b^	−0.01 (−0.05, 0.03) ^c^	−0.06 (−0.12, 0.00) ^d^	**0.005**	0.192	Linear
	2	0.04 (−0.02, 0.10)	0.01 (−0.03, 0.06)	−0.01 (−0.05, 0.03)	−0.04 (−0.10, 0.02)	0.057	0.195	
	3	0.05 (−0.01, 0.11) ^a^	0.02 (−0.02, 0.06) ^b^	−0.01 (−0.05, 0.03) ^c^	−0.05 (−0.11, 0.01) ^d^	**0.021**	0.219	
	4	0.05 (−0.01, 0.11) ^a^	0.02 (−0.02, 0.06) ^b^	−0.01 (−0.05, 0.03) ^c^	−0.05 (−0.11, 0.01) ^d^	**0.025**	0.254	
Letter verbal fluency	1	0.05 (−0.01, 0.11) ^a^	0.02 (−0.03, 0.06) ^b^	−0.01 (−0.05, 0.03) ^c^	−0.05 (−0.11, 0.01) ^d^	**0.021**	0.737	Linear
	2	0.02 (−0.03, 0.07)	0.01 (−0.03, 0.05)	0.00 (−0.04, 0.03)	−0.02 (−0.08, 0.03)	0.284	0.643	
	3	0.03 (−0.04, 0.09)	0.02 (−0.03, 0.06)	0.00 (−0.05, 0.05)	−0.04 (−0.09, 0.02)	0.082	0.648	
	4	0.03 (−0.04, 0.09)	0.01 (−0.03, 0.06)	0.00 (−0.05, 0.05)	−0.03 (−0.09, 0.02)	0.102	0.741	
Mini-Mental State Examination	1	0.02 (−0.05, 0.09)	−0.03 (−0.09, 0.04)	0.04 (−0.02, 0.09)	0.00 (−0.07, 0.08)	**<0.001**	**0.006**	Nonlinear 4 knots
	2	0.00 (−0.07, 0.06) ^a,b^	−0.03 (−0.09, 0.03) ^a^	0.04 (−0.01, 0.09) ^b^	0.02 (−0.05, 0.09) ^a,b^	**0.004**	**0.007**	
	3	0.02 (−0.05, 0.08)	−0.03 (−0.09, 0.03)	0.03 (−0.02, 0.08)	0.01 (−0.06, 0.08)	**0.002**	**0.010**	
	4	0.02 (−0.05, 0.08)	−0.03 (−0.09, 0.03)	0.03 (−0.02, 0.08)	0.01 (−0.06, 0.08)	**0.003**	**0.015**	

* Higher cognitive scores indicate better performance except in the case of power of attention and speed of memory, where higher scores indicate slower speed and worse performance. ^ Model 1: De-seasonalised serum 25OHD only, Model 2: Model 1 plus age and estimated IQ, Model 3: Model 2 plus BMI, alcohol consumption, smoking status, physical activity (low, medium, high), sitting hours per day, employment status (employed, retired or other), and use of vitamin D supplements, Model 4: Model 3 plus self-reported health status and history (yes vs. no) of hypertension, cardiovascular disease, diabetes, depression, and anxiety. # *p*-Value overall: overall *p*-value for serum 25OHD where nonlinear model was selected, or the *p*-value for the linear term where linear model was selected; *p*-value nonlinear: *p*-value from likelihood ratio test of whether nonlinear model improves on the simple, linear model. *p*-Values ≤ 0.05 are highlighted in bold. ^a–d^ In rows with superscripts (a, b, c, d), mean values without a common letter indicate that means differ, *p* < 0.05.

## Data Availability

Application for access to the deidentified, individual BHAS participant data underlying the results of this study can be made to the BPMRI. Applications would be reviewed by the BPMRI Scientific Committee.

## Supplementary Materials

The following are available online at https://www.mdpi.com/article/10.3390/ijerph19010450/s1: Table S1. Akaike information criteria (AIC) comparing regression models for cognitive *z*-scores that include restricted cubic splines (RCS) with 3–6 knots for serum 25OHD level; Table S2. Characteristics of Busselton Healthy Ageing Study (BHAS) women and men across sex-specific serum 25OHD quartiles; Table S3. Cognitive *z*-scores (least square means and 95% CLs) at mid-quartile levels of de-seasonalised serum 25OHD in BHAS women not taking vitamin D supplements, derived from ‘best-fit’ of linear and nonlinear (RCS) and fully adjusted models (Model 4); Table S4. Cognitive *z*-scores (least square means and 95% CLs) at mid-quartile levels of de-seasonalised serum 25OHD in BHAS men not taking vitamin D supplements, derived from ‘best fit’ of linear and nonlinear (RCS) and fully adjusted models (Model 4).
