# Supplementary material for: Investigating Potential Dose–Response Relationships between Vitamin D Status and Cognitive Performance: A Cross-Sectional Analysis in Middle- to Older-Aged Adults in the Busselton Healthy Ageing Study
Source: Int J Environ Res Public Health. 2021 Dec 31;19(1):450. doi: 10.3390/ijerph19010450 (PMC8744852; doi:10.3390/ijerph19010450)
Supplement: Supplementary file 1 [file ijerph-19-00450-s001.zip › ijerph-1478394-supplementary.pdf]

Supplementary Table S1. Akaike information criteria (AIC) comparing regression models for cognitive *z*-scores that include restricted cubic splines (RCS) with 3–6 knots for serum 25OHD level.

Cognitive *z*-score	Model	Women				Men			
		n = 3	*n* = 4	*n* = 5	*n* = 6	*n* = 3	*n* = 4	*n* = 5	*n* = 6
Continuity of attention factor	1	7597	7599	7601	7601	6229	6229	6231	6233
	2	7418	7420	7422	7421	6102	6103	6105	6107
	3	7406	7407	7409	7408	6090	6090	6092	6094
	4	7383	7385	7386	7385	6073	6074	6075	6077
Power of attention factor	1	7605	7607	7607	7609	6230	6232	6234	6235
	2	7441	7443	7443	7445	6081	6082	6084	6086
	3	7437	7439	7439	7441	6078	6079	6081	6083
	4	7441	7443	7442	7445	6082	6083	6085	6087
Quality of working memory factor	1	7602	7603	7602	7604	6232	6234	6235	6237
	2	7474	7475	7474	7476	6147	6149	6150	6153
	3	7488	7490	7489	7490	6137	6138	6139	6141
	4	7475	7476	7475	7477	6145	6147	6148	6150
Quality of episodic memory factor	1	7606	7607	7606	7608	6227	6229	6231	6227
	2	7331	7332	7331	7333	6005	6006	6008	6006
	3	7340	7341	7339	7341	6004	6006	6007	6005
	4	7351	7353	7351	7353	6010	6012	6014	6012
Speed of memory factor	1	7601	7603	7605	7607	6228	6229	6229	6229
	2	7359	7361	7363	7364	6068	6069	6070	6071
	3	7306	7308	7310	7311	6073	6075	6075	6075
	4	7310	7312	7314	7316	6082	6084	6085	6085
Semantic verbal fluency	1	7605	7606	7605	7607	6223	6225	6226	6226
	2	7179	7181	7180	7182	6012	6014	6016	6016
	3	7185	7187	7186	7188	6033	6035	6037	6037
	4	7185	7186	7186	7188	6045	6047	6049	6050
Letter verbal fluency	1	7606	7607	7603	7604	6228	6230	6232	6233
	2	7032	7033	7030	7030	5682	5684	5685	5687
	3	7043	7045	7041	7042	5677	5679	5680	5682
	4	7052	7053	7049	7050	5676	5679	5680	5682
Mini-Mental State Examination	1	7605	7607	7609	7611	6221	6215	6217	6219
	2	7092	7094	7095	7097	5838	5833	5834	5836
	3	7075	7077	7078	7080	5830	5824	5826	5828
	4	7057	7059	7060	7062	5835	5830	5832	5834

Model 1: De-seasonalised serum 25OHD only, Model 2: Model 1 plus age and estimated IQ, Model 3: Model 2 plus BMI, alcohol consumption, smoking status, physical activity (low, medium, high), sitting hours per day, employment status (employed, retired or other), and use of vitamin D supplements, Model 4: Model 3 plus self-reported health status and history of hypertension, cardiovascular disease, diabetes, depression, and anxiety.

SENSITIVE

Supplementary Table S2. Characteristics of Busselton Healthy Ageing Study (BHAS) women and men across sex-specific serum 25OHD quartiles.

	**Women**				**Men**			
	**Q1**	**Q2**	**Q3**	**Q4**	**Q1**	**Q2**	**Q3**	**Q4**
	**(*N* = 670)**	**(*N* = 669)**	**(*N* = 670)**	**(*N* = 669)**	**(*N* = 548)**	**(*N* = 549)**	**(*N* = 548)**	**(*N* = 549)**
Age (years)	57.2 ± 5.8	58.1 ± 5.5	58.0 ± 5.9	58.2 ± 5.7	57.8 ± 5.7	58.1 ± 6.0	58.1 ± 5.8	58.3 ± 6.0
Body mass index (BMI)	29.8 ± 6.0	28.5 ± 5.3	27.4 ± 5.0	26.0 ± 5.0	29.7 ± 4.8	28.7 ± 4.2	28.3 ± 3.7	27.3 ± 3.4
Education—completed tertiary, *n* (%)	123 (18.4)	139 (20.8)	136 (20.3)	141 (21.1)	117 (21.4)	108 (19.7)	115 (21.0)	80 (14.6)
Smoking status, *n* (%)								
Never	316 (47.2)	340 (50.8)	346 (51.6)	348 (52.0)	242 (44.2)	236 (43.0)	233 (42.5)	220 (40.0)
Ex	272 (40.6)	260 (38.9)	278 (41.5)	284 (42.5)	235 (42.9)	255 (46.4)	258 (47.0)	266 (48.5)
Current	82 (12.2)	69 (10.3)	46 (6.9)	37 (5.5)	71 (12.9)	58 (10.6)	57 (10.4)	63 (11.5)
Alcohol consumption (glasses per week), *n* (%)								
Nil	80 (11.9)	70 (10.5)	55 (8.2)	51 (7.6)	43 (7.8)	33 (6.0)	34 (6.2)	23 (4.2)
0 to 2.5	248 (37.0)	218 (32.6)	200 (29.9)	177 (26.5)	85 (15.5)	78 (14.2)	73 (13.3)	50 (9.1)
2.6 to 8.5	148 (22.1)	187 (28.0)	192 (28.7)	188 (28.1)	98 (17.9)	105 (19.1)	97 (17.7)	86 (15.7)
8.6 to 17.9	132 (19.7)	127 (19.0)	160 (23.9)	178 (26.6)	113 (20.6)	139 (25.3)	118 (21.5)	139 (25.3)
18+	62 (9.3)	67 (10.0)	63 (9.4)	75 (11.2)	209 (38.1)	194 (35.3)	226 (41.2)	251 (45.7)
Physical activity category (MET minutes per week), *n* (%)								
0–599	207 (30.9)	160 (23.9)	152 (22.7)	115 (17.2)	113 (20.6)	84 (15.3)	76 (13.9)	57 (10.4)
600–2999	262 (39.1)	270 (40.4)	273 (40.7)	279 (41.7)	181 (33.0)	174 (31.7)	161 (29.4)	141 (25.7)
3000+	201 (30.0)	239 (35.7)	245 (36.6)	275 (41.1)	254 (46.4)	291 (53.0)	311 (56.8)	351 (63.9)
Employment status, *n* (%)								
Employed	443 (66.1)	407 (60.8)	367 (54.8)	350 (52.3)	423 (77.2)	409 (74.5)	395 (72.1)	376 (68.5)
Retired	130 (19.4)	159 (23.8)	186 (27.8)	205 (30.6)	80 (14.6)	100 (18.2)	114 (20.8)	133 (24.2)
Other	97 (14.5)	103 (15.4)	117 (17.5)	114 (17.0)	45 (8.2)	40 (7.3)	39 (7.1)	40 (7.3)

SENSITIVE

	Women				Men			
	**Q1**	**Q2**	**Q3**	**Q4**	**Q1**	**Q2**	**Q3**	**Q4**
	**(*N* = 670)**	**(*N* = 669)**	**(*N* = 670)**	**(*N* = 669)**	**(*N* = 548)**	**(*N* = 549)**	**(*N* = 548)**	**(*N* = 549)**
Medical histories, *n* (%)								
Depression	160 (23.9)	164 (24.5)	148 (22.1)	124 (18.5)	84 (15.3)	87 (15.8)	89 (16.2)	75 (13.7)
Anxiety	50 (7.5)	38 (5.7)	40 (6.0)	30 (4.5)	15 (2.7)	28 (5.1)	14 (2.6)	15 (2.7)
CVD	32 (4.8)	19 (2.8)	21 (3.1)	26 (3.9)	50 (9.1)	44 (8.0)	39 (7.1)	45 (8.2)
Diabetes	55 (8.2)	41 (6.1)	34 (5.1)	31 (4.6)	56 (10.2)	42 (7.7)	41 (7.5)	16 (2.9)
Hypertension	290 (43.3)	255 (38.1)	242 (36.1)	236 (35.3)	270 (49.3)	270 (49.2)	227 (41.4)	221 (40.3)
Self-reported health status, *n* (%)								
Poor/fair	82 (12.2)	47 (7.0)	48 (7.2)	41 (6.1)	67 (12.2)	56 (10.2)	46 (8.4)	41 (7.5)
Good	282 (42.1)	260 (38.9)	238 (35.5)	206 (30.8)	252 (46.0)	236 (43.0)	209 (38.1)	210 (38.3)
Very good/excellent	306 (45.7)	362 (54.1)	384 (57.3)	422 (63.1)	229 (41.8)	257 (46.8)	293 (53.5)	298 (54.3)
Sitting hours per day	4.5 ± 2.8	4.4 ± 2.5	4.1 ± 2.3	4.3 ± 2.4	5.1 ± 2.9	4.8 ± 2.6	4.6 ± 2.6	4.4 ± 2.5
De-seasonalised serum 25OHD (nM/L)	51.5 ± 7.7	68.6 ± 3.7	83.0 ± 4.5	109.9 ± 20.6	58.0 ± 7.9	75.3 ± 4.0	89.1 ± 4.1	116.9 ± 21.4
Use of vitamin D supplements, *n* (%)	56 (8.4)	101 (15.1)	141 (21.0)	137 (20.5)	30 (5.5)	31 (5.7)	31 (5.7)	43 (7.8)
Estimated IQ	102.8 ± 9.7	102.5 ± 9.7	102.9 ± 9.4	102.3 ± 9.7	102.9 ± 11.1	101.9 ± 11.2	102.2 ± 11.1	101.2 ± 9.9

Values shown are means ± standard deviations unless otherwise indicated; percentages (%) may not always add up to 100 due to rounding. MET, metabolic equivalent, a measure of physical activity related to metabolic rate; CVD, cardiovascular disease.

SENSITIVE

Supplementary Table S3. Cognitive *z*-scores (least square means and 95% CLs) at mid-quartile levels of de-seasonalised serum 25OHD in BHAS women not taking vitamin D supplements, derived from 'best fit' of linear and nonlinear (RCS) and fully adjusted models (Model 4)

Cognitive *z*-scores *	Quartile 1 (51.9 nM/L)	Quartile 2 (67.3 nM/L)	Quartile 3 (81.4 nM/L)	Quartile 4 (102.8 nM/L)	*p*-Values # Overall	Nonlinear
CoA	-0.11 (-0.11, 0.04) [a]	-0.01 (-0.06, 0.04) [b]	0.04 (-0.01, 0.10) [c]	0.05 (-0.01, 0.11) [c]	**0.005**	**0.019**
PoA	-0.03 (-0.09, 0.03)	-0.02 (-0.07, 0.02)	-0.02 (-0.06, 0.02)	-0.02 (-0.08, 0.04)	0.839	0.067
QoWM	-0.04 (-0.10, 0.02)	-0.02 (-0.07,0.02)	0.00 (-0.05, 0.04)	0.02 (-0.04, 0.08)	0.169	0.384
QoEM	0.03 (-0.03, 0.09)	0.01 (-0.03, 0.06)	0.00 (-0.04, 0.04)	-0.02 (-0.08, 0.04)	0.314	0.291
SoM	-0.02 (-0.08, 0.03)	-0.01 (-0.05, 0.03)	0.00 (-0.04, 0.04)	0.02 (-0.03, 0.08)	0.267	0.722
SVF	0.01 (-0.05, 0.07)	-0.01 (-0.05, 0.04)	-0.02 (-0.06, 0.02)	-0.04 (-0.10, 0.02)	0.298	0.299
LVF	-0.02 (-0.08, 0.03)	-0.01 (-0.05, 0.03)	0.01 (-0.03, 0.04)	0.03 (-0.03, 0.08)	0.246	0.440
MMSE	-0.04 (-0.09, 0.02)	-0.02 (-0.06, 0.02)	0.00 (-0.04, 0.04)	0.03 (-0.02, 0.09)	0.092	0.799

* Higher cognitive scores indicate better performance except in the case of power of attention and speed of memory, where higher scores indicate slower speed and worse performance. CoA, continuity of attention; PoA, power of attention; QoWM, quality of working memory; QoEM, quality of episodic memory; SoM, speed of memory; SVF, semantic verbal fluency; PVF, letter verbal fluency; MMSE, Mini-Mental State Examination.

Model 4: De-seasonalised 25OHD, age, estimated IQ, BMI, alcohol consumption, smoking status, physical activity (low, medium, high), sitting hours per day, employment status (employed, retired or other), self-reported health status and history (yes vs. no) of hypertension, cardiovascular disease, diabetes, depression, and anxiety.

p-Value overall: overall *p*-value for serum 25OHD where nonlinear model was selected, or the *p*-value for the linear term where linear model was selected; *p*-value nonlinear: *p*-value from likelihood ratio test of whether nonlinear model improves on the simple, linear model.

[a–d] In rows with superscripts (a, b, c, d), mean values without a common letter indicate that means differ, $p < 0.05$.

SENSITIVE

Supplementary Table S4. Cognitive *z*-scores (least square means and 95% CLs) at mid-quartile levels of de-seasonalised serum 25OHD in BHAS men not taking vitamin D supplements, derived from 'best fit' of linear and nonlinear (RCS) and fully adjusted models (Model 4)

Cognitive *z*-scores *	Quartile 1 (51.9 nM/L)	Quartile 2 (67.3 nM/L)	Quartile 3 (81.4 nM/L)	Quartile 4 (102.8 nM/L)	*p*-Values #	
					Overall	Nonlinear
CoA	-0.05 (-0.11, 0.02) [a]	-0.02 (-0.06, 0.03) [b]	0.01 (-0.03, 0.05) [c]	0.05 (-0.01, 0.11) [d]	**0.034**	0.859
PoA	-0.03 (-0.09, 0.04)	-0.02 (-0.06, 0.03)	-0.01 (-0.05, 0.03)	0.01 (-0.06, 0.07)	0.499	0.125
QoWM	0.01 (-0.06, 0.07)	0.00 (-0.05, 0.04)	-0.01 (-0.06, 0.03)	-0.03 (-0.09, 0.04)	0.525	0.323
QoEM	-0.04 (-0.11, 0.04)	0.02 (-0.03, 0.07)	0.03 (-0.02, 0.09)	-0.01 (-0.07, 0.06)	0.094	**0.031**
SoM	-0.03 (-0.09, 0.03)	-0.01 (-0.06, 0.03)	0.00 (-0.04, 0.05)	0.03 (-0.03, 0.09)	0.172	0.257
SVF	0.06 (0.00, 0.12) [a]	0.02 (-0.03, 0.06) [b]	-0.01 (-0.06, 0.03) [c]	-0.07 (-0.13, 0.00) [d]	**0.009**	0.164
LVF	0.03 (-0.04, 0.09)	0.01 (-0.03, 0.05)	-0.01 (-0.06, 0.04)	-0.04 (-0.10, 0.02)	0.108	0.875
MMSE	0.01 (-0.06, 0.08)	-0.04 (-0.10, 0.03)	0.02 (-0.03, 0.08)	0.00 (-0.07, 0.07)	**0.003**	**0.016**

* Higher cognitive scores indicate better performance except in the case of power of attention and speed of memory, where higher scores indicate slower speed and worse performance. CoA, continuity of attention; PoA, power of attention; QoWM, quality of working memory; QoEM, quality of episodic memory; SoM, speed of memory; SVF, semantic verbal fluency; PVF, letter verbal fluency; MMSE, Mini-Mental State Examination.

Model 4: De-seasonalised 25OHD, age, estimated IQ, BMI, alcohol consumption, smoking status, physical activity (low, medium, high), sitting hours per day, employment status (employed, retired or other), self-reported health status and history (yes vs. no) of hypertension, cardiovascular disease, diabetes, depression, and anxiety.

p-Value overall: overall *p*-value for serum 25OHD where nonlinear model was selected, or the *p*-value for the linear term where linear model was selected; *p*-value nonlinear: *p*-value from likelihood ratio test of whether nonlinear model improves on the simple, linear model.

[a–d] In rows with superscripts (a, b, c, d), mean values without a common letter indicate that means differ, $p < 0.05$.

SENSITIVE
